# The Detrimental Physical Properties of Expansion and Compression of Plaster of Paris

**Published:** 1907-01-15

**Authors:** J. H. Prothero

**Affiliations:** Chicago


### THE DETRIMENTAL PHYSICAL PROPERTIES OF EXPANSION AND COMPRESSION OF PLASTER OF PARIS.

BY J. H. PROTHERO, D.D.S., OF CHICAGO.

Read before the Michigan State Dental Association, July 10th, 1906.

For some time past I have been interested in the peculiar physical properties of plaster of paris, particularly those of expansion and compression.

To study this material with any degree of accuracy, it was necessary to construct suitable recording instruments for noting the changes which ordinarily occur, as well as those brought about by manipulation procedures. The results of many experiments conducted with the appliances mentioned have been given to the profession from time to time in various papers and Journal articles. It is not possible at this time to present anything new in this field, but an effort will be made to point out the results of expansion, warpage, and compression on denture adaptation.

### EXPANSION.

When dental plaster of a good quality and water are mixed in proper proportions, re-crystallization or setting usually begins in from two to six minutes. At the initial stage of the setting process a very slight amount of contraction is noticable: then follows a short period of inertia lasting a minute or two after which expansion sets in.

The expansion begins slowly, increases rapidly until in about four or five minutes the maximum rate of movement is reached, when it gradually decreases and is apparently over in about twenty minutes from the time of making the mix. As a matter of fact, expansion to a limited extent continues for twenty-four hours or even a longer time with no perceptible contraction following. During the setting process a rise of about ten degrees in temperature in the mass is noticable.

Considerable difference of opinion exists as to the percentage of expansion which ordinarily occurs. Dr. Spence states that the average is about one percent, while in the experiments I conducted the average was about one-fourth of one percent. Without doubt the difference noted can be partially accounted for by the difference in manipulation.

In the experiments alluded to an effort was made to subject the material to the same treatment it ordinarily receives in the dental laboratory. No attempt was made to induce abnormal movement which could easily have been brought about by long continued and rapid stirring, and I am therefore of the opinion that my own estimate is more nearly correct. The fact remains, however, that all plasters expand whether much or little, and that this movement even under the most favorable conditions, results in enlarged and frequently warped molds.

### WARPAGE

Warpage of plaster models is induced in two ways: First, as the result of expansion, and second, as the result of compression in flask closure.

Let us for a moment consider the result of expansion of a plaster upper impression in a metallic tray with fixed sides.

The sides of the tray being immovable, act as points of resistance and prevent lateral expansion. The palatine portion being arched, is unfavorable for holding the impression closely to this area, and consequently as expansion occurs, the impression is lifted away from the tray by the expansion force.

This fact is denied by some, but can easily be demonstrated by trimming the over-hanging plaster away from the distal margin of the tray when the space between the impression and the tray will be brought into view. A model secured from such an impression would have a deeper palatine arch than the mouth it represents, while if the tray and impression were not quickly removed from such a model, the latter would also warp in the same location and the error thus be practically doubled. A denture moulded over such a model would touch the central palatine portion of the mouth before becoming firmly seated on the alveolar border, and therefore would tip under the slightest stress.

A few suggestions relative to obviating this trouble are here offered.

Add a little sulphate of potassium to the water before introducing the plaster to accelerate the setting and also to reduce the tendency to expand. Stir the mass very little or just enough to insure a uniform mix.

Trim the impression immediately upon removal from the mouth, apply the separating medium and introduce the plaster for the model. When the latter has set sufficiently to permit, remove the tray and impression. This will prevent warpage to a great extent, but will not obviate expansion. The expansion is compensated for by scraping the periphery of the model along the line where the denture margin will rest. These steps if properly carried out will result in good denture adaptation, if in the subsequent step of closing the packed rubber case the model is not warped or compressed by pressure.

### COMPRESSIBILITY.

Plaster is capable of being condensed if sufficient pressure is brought to bear upon it. Condensation occurs in line of direction of the applied force, and no where else. The molecules lying just outside of the line of direction of the force are not disturbed by the molecules subjected to pressure since there is no tendency to slide or creep from under the load and therefore no bulging of adjacent surfaces occurs.

A model imbedded in a flask that contains an excessive amount of vulcanizable rubber will be compressed and warped out of shape under undue pressure. The force capable of being applied by flask closing devices is enormous. Take for instance a flask wrench four inches in length and a flask bolt having 20 threads to the inch. Apply a power of 25 lbs. on the wrench handle and the result is a pressure of over two tons on the contents of the flask.

The rule for finding this is as follows: Power is to weight as the distance between two contiguous threads is to the circumference of the circle described by the wrench at the point of application of force. Usually one-third is deducted for friction.

I am convinced that much more force is usually applied in flask closing than plaster models are capable of withstanding without changing form or distorting, and this fact is responsible for many of the failures which occur.

The object in carrying out the steps that I have outlined is to preserve the integrity of the plaster model. In other words, when plaster models are subject to moisture they are rendered softer and under ordinary pressure will compress more readily than when dry, therefore, if it is possible for you to carry out the steps of packing and closing the flask without moistening the plaster, you will get better results.

Another point to vulcanize in steam vapor. A cubic inch of water makes a cubic foot of steam, and the plan that I have followed is to place in the bottom of the vulcanizer a small block of zinc and place the flask on that and place enough water in the vulcanizer to steam the interior, thus placing the flask entirely above the water. The object in using the zinc is to prevent the flask becoming blackened with the iron sulphide which so readily occurs. The reaction which occurs in the vulcanizing takes place with the zinc instead of with the iron or brass flask.

By strengthening the periphery of the model we get better results than we would were is done by the usual methods. There is not one vulcanized case in five-thousand that is distorted where perfect adaptation is secured. A force of from one-half to twenty pounds will displace any denture that most of us have ever seen.

DISCUSSION.

Dr. N. S. Hoff, Ann Arbor: The statements made in this paper are so obviously conclusive that it leaves one no ground for criticism, and I have no disposition to criticise at any rate. In regard to this matter of expansion of plaster impressions, due to the setting of the plaster. Take a plaster impression in a smooth polished tray and leave it several hours over night, in a warm room and you will find it is very easy to lift it out of the tray. I think the essayist is perfectly justified in saying there is a change in the form of the impression. I used to think the model had contracted but now I can see from the experiment the essayist has made that it is probably an expansion. I am hardly convinced that this expansion is of sufficient amount to badly distort the impression. It would appear to me that the impression should expand in all directions so that the amount of change was more or less equalized. It has never occured to me before that faulty adaptation of vulcanized dentures was due to the fact that there was a change in the form of the plaster impression itself. I have always attributed this to faulty manipulation in the vulcanizing process; to the methods used in packing and vulcanizing the rubber, more than to the change of form in the plaster impression or model upon which it was made. My favorite method of taking impressions is with plaster, unless there are inequalities in the density of the tissues requiring condensation, when I want a beeswax or modeling compound impression. Usually I take a plaster impression preferably because it gives me not only the correct form of the mouth but the absolute detail, more than you can get with modeling compound or any other impression material. I usually pour my impression very soon after it is taken and do not use the separating medium as many do, simply soaking the impression in water before pouring

Place the impression directly in water and allow it to take up all the water it will, until there is no appearance of any bubbles coming up through the water. I mix my plaster by first dissolving the potash in the water, then sifting the plaster in until the water is thoroughly full of plaster, let it stand a moment and then pour off the free water on top and stir it just a little, enough to mix the plaster and pour it at once. I get a hard model in this way that does not break during manipulation, and I feel that it does not condense under the pressure that is necessary in packing and vulcanizing. Many times we blunder by having the model too thin. We cut the model down, particularly where there is a high arch, until there is not enough substance to it to sustain the pressure brought upon it.

In the preparation of the models themselves for the vulcanizing process we often make the mistake of saturating them with water after we have secured the model, or in boiling out the wax. We ought never to do this, a plaster model that is very hard and dense if simply dropped into water for thirty seconds and taken out can be whittled very easily. If you have a very thin model it will soak up enough water in a short time to sufficiently destroy its integrity to cause it to break under the pressure put upon it in closing the flask.

In the matter of flasking these pieces, I have not yet found a flask that is at all to my notion. All of our flasks are not deep enough. You cannot put into any of the brass flasks on the market an ordinary model with the teeth on it and have more than perhaps half an inch of plaster over the palate where the great amount of pressure is usually brought to bear. Sometimes models here are cut down to possibly one-eight of an inch, so that you can hold them up to the light and almost see through them. You cannot expect a model of that kind to stand the enormous pressure of two tons that Dr. Prothero says is frequently put upon such a model, without its fracturing. The support that you get from the investing plaster is not sufficient to support such a thin model. If the model could be made thick enough it would withstand the pressure without giving way. The flasks should be at least 3-4 inches deeper than they are.

In connection with packing the flask another thing might be mentioned. We usually close them in boiling water and put in so much rubber that we cannot possibly get the flask together, and then we boil for ten or twenty minutes to get the vulcanite to flow away in order to get the flask together.

This boiling of the plaster must disintegrate it and destroy its integrity.

In the matter of cutting gateways in the investment we often make a mistake. The ordinary method of simply cutting grooves from the matrix to the rim of the flask is a mistake. A better plan is the method advocated by Dr. Snow, which is to cut a groove in the plaster investment all the way around the flask at its outer edge, cutting it deep towards the flask and beveling it to the edge of the matrix so that as the flask goes together there is plenty of space for all the excess of vulcanite to flow out, but when the flask is entirely closed the two edges of the investment come in contact and no more of the vulcanite can escape. During the process of heating the vulcanite swells and expands until you get to the vulcanizing point where it begins to carbonize and harden, and then it begins to shrink upon itself. If you have gateways that carry off the excess of vulcanite it never gets any pressure, because the vulcanite flows away and never gets back into the matrix, and as the vulcanite in the mold shrinks, it draws away from the model or from the teeth leaving spaces about the teeth.

In this form of investment we should use the Donham Spring Clamp to hold the flasks together, as the pressure, of course, must be enormous if we use the ordinary bolts for holding the flask together. As the vulcantie swells in the process of heating these springs allow the flask to open, and as it hardens it comes back together. In this way we get the vulcanite hardened in the most perfect manner, and avoid its shrinking away from the teeth. We should vulcanize all rubber under spring pressure and get rid of the enormous pressure that we would otherwise subject the plaster mould to if we have only the bolts and screws. We do not pack vulcanite with the care we should. We do not even go to the trouble of warning the flask, and we cut great strips of vulcanite and put them in the flask cold, and try to screw them together in boiling water and expect to get good results. It is remarkable that we get as good results as we do from this slovenly method. The fact that we get any desirable results from the process it seems to me is a great compliment to the vulcanite, demonstrating that it is an exceedingly tractable material and can withstand a great amount of abuse without resenting it.

If we would take more pains we should get more sanitary plates, and I have no doubt that we should be surprised that our vulcanite plates fit so much better than they did when we did not take into account these methods. A large number of dentists make vulcanite plates, probably many more of them than of any other kind. We ought to make them in the best possible manner or not at all Because they can be made so easily they are often done by the office boy or some equally inexperienced or unintelligent person. It is entirely unprofessional for us not to study the details of this work and do it as it should be done.

Dr. Land: If we cannot determine the different angles of the model surfaces we cannot arrive at any conclusion as to which way the shrinkage or expansion of the model will take. If a high model is packed in the center and you don't provide for the relief of the excessive pressure of the vulcanite it is going to push the center out of place when you pack it. If every model was perfectly flat or horizontal it would not matter if there were twenty tons on it, it would be exactly the same shape, so we must study the angles. If every mouth was two inches square and perfectly flat, where would your changes come? Where a model is at different angles and you pack on those angles and do not take into consideration the direction of the flow according to the angles you will never arrive at any satisfactory conclusion until that influence is accounted for. I never find it necessary to cut a gate in the plaster mould because I put the mass of vulcanite on the alveolar ridge and let the center be the relief. There is where our trouble comes. If you make all your pressure on the alveolar arch it will not do any particular harm because you will get relief at the center. If you put no rubber in the center and let the force come on the alveolar ridge, you will have no force on the center. If every arch you made simply described a horizontal plane, expansion would amount to nothing. If it shrank only one per cent it would amount to nothing in an artificial set of teeth, because one per cent will always conform in any mouth to any angle.

It would conform if there was a change of a quarter of an inch. You must consider these angles, or you will always be at sea.

Dr. C. H. Worboys, Albion: The expansion of plaster is readily shown to any one who works in the plaster if he makes the model flat. I will give you a little experience I have had. Out of seven plaster impressions taken of five different mouths, dies poured in low fusing metal resulted in five misfits. Five plaster impressions of the same five mouths, taken as most of us take these plaster models, zinc dies made from those plaster models resulted in five fits, that is, the patients could wear them. We all know that zinc shrinks considerably and the shrinkage of the zinc just about made up for the expansion of the plaster.

Speaking of the plaster expanding in the tray—it just occurred to me that perhaps the tray expanded from the heat somewhat; most metals do expand, and it may have a tendency to warp the plaster impression which is not as fully set at that time as it would be later.

Speaking of having deep flasks, I do not take any stock in that at all for a plate with a high arch. If we had a mouth to be fitted with a plate that was very flat we should get a thick body of plaster and that would give us a chance for greater compression than a thin body of plaster .

You cannot compress a quarter or an eighth of plaster as much as you can a half an inch, so that part I do not think makes any difference.

About the vulcanite coming up tight to the teeth, I think that the large waste gates help to let it away. Some of the best results that I have had in plate work has been when there was no scraping of the models or impressions. Why I succeeded so well in these cases and failed so utterly in others I am not able to say.

Dr. Prothero: I am still of the same opinion that I was before that waset gates are valuable where made properly, and Dr. Hoff has outlined the method that I have followed and for which he gave credit to Dr. Snow for having mentioned. I think I got the idea of carving at the margin, so that when the flask is partly closed you have free scope for excess. I can get just as good adaptation by packing the flask as I have outlined as any one can in any other way. If you cut the gates right and use slow pressure and plenty of dry heat you will get the surplus out and into the waste space without distorting the face of the model a bit. I have had better results thant I ever dreamed of having, by following the old methods, since following the methods I have given you tonight. Instead of vulcanizing work being a bugbear it will be a pleasure, because your dentures will fit comparatively well when placed in the mouth. I do not think I can possibly get perfect adaptation.

I believe that if we secured perfect adaptation the patient would not be able to wear the plates with any comfort. If the denture pressed all over the mouth with the pressure of 50 pounds to 60 pounds it would be very uncomfortable to wear. Any manipulation which tends to enlarge, reduce or warp a model will interfere with securing perfect adapta tion. I do not care whether it is expansion, compression or warpage, no matter how slight, if we can get rid of it we shall get better results.

I want to outline a method of taking impressions, which I have followed with a great deal of satisfaction. It is not supposed to be very popular but I am coming to try it in certain cases. When there are no teeth in the mouth a modeling compound impression can be secured which will give a more accurate representation of the mouth than plaster of paris will give. Take the impression in the ordinary way, using good modeling compound. Chill it all through. If you use cold water to chill it, wipe off the water and hold it over a small alcohol flame or Bunsen burner and warm the surface to 1-16 to 1-24 of an inch in depth until the surface is quite plastic and the body of the impression is stiff. Introduce it in the mouth and press it firmly to place and you will find in many cases it will be almost impossible to remove the impression the second time. The best qualtities of modeling compound will not expand or contract to any appreciable extent. Remember, now, I am not advocating the use of compound for partial cases or where there are heavy undercuts in the ridge but in cases where the impression can be taken away readily.

A word in regard to the use of sulphate of potassium. In the experiments conducted I found that about 5-10 of a gram of sulphate of potassium in 40 c. c. of water would require about 55 dr. plaster of paris to make and mix without throwing out any water, and the mass would be of medium consistency suitable for taking impression, and then if it was stirred enough to mix the water and plaster you would get the minimum amount of expansion.

I have a machine for measuring the expansion which reads to 1-10,000 of an inch, which is as accurate as any machine: it is not a complicated machine either. When plaster of paris mixed in the manner I have just described is placed in that machine it shows only from one to fifteen ten thousandths of an inch expansion, while if stirred for half a minute without the addition of sulphate of potassium it will show from 150 to 200 ten thousandths. That will reduce it to the minimum so that we will have little difficulty arising from the expansion which might occur, while if we follow the ordinary method of stirring we get grave results and results detrimental to adaptation. Sulphate of potassium should not be used in plaster that is used for models for the reason that steam and water attack a model containing sulphate of potassium much more rapidly than if the plaster is mixed without potassium, therefore, we are trying to keep the plaster model hard as long as possible until the rubber has begun to carbonize.

A friend of mine told me one day what kind of a press he used and the pressure he put on the end of the handle, and I sat down and figured it out for him that he was using 7 tons for closing eack flask. You would be surprised at the enormous pressure we ordinarily use with the ordinary four inch flask wrench. The double end handle flask wrench will exert a pressure of two to three tons, and the Donham spring, will I believe, if handled properly give better results than the closing of flask under heavy pressure, but will not give any better results than can be secured by using a moderate amount of pressure when your models are dry. Nearly all of you, I believe, have put sufficient pressure on models in closing to fracture the models. I know I have, and I know a great many others who have, as the plaster was not of sufficient density to hold up the model, in places under the heavy pressure to which it is subjected.

In regard to adaptation in general I will say that we owe it to the vulcanite largely, but more largely to mother Nature for having covered the Palatine portion of the mouth with a tough mucous membrane as Dr. Land suggests, that membrane will in time be drawn down into the heart shaped suction chambers that we put into vulcanite dentures. If we can secure a perfect impression of the mouth and mould our plate on its model without destroying it we are going to get a more perfect adaptation to the tissues than if we depend on a distorted model.

Tomorrow, I will have three instruments which have been constructed purposely to show the movements in setting plaster, and then you will readily see that some of the things I have mentioned tonight are true and not mere theory.

The question was asked whether lime water has any effect on controling expansion. It has not one particle of effect on it. I have made tests and found out that lime water has no effect whatever on controling expansion.

Dr. P. F. Hines: I was very glad that the doctor brought up the matter of compression. I have read a series of articles in the Items of Interest of '94. I thought them very interesting, and have been endeavoring to put their conclusion into practice especially in the matter of packing and using dry heat in accordance with the ideas our essayist has suggested. I believe in packing dry, as the results are better, and I think by so doing we certainly get rid of that matter of compression in the deep arches, the alveolar ridge being so much more plastic than it is in the palatine surface that it gives more chance for compression. Over the palatine surface there is, we will say 3-4 in. of plaster when invested in the flask and the alveolar ridge is from 1-4 to 3-4 in. thick, there will be therefore greater compression along the line of the alveolar ridge than on the palatine surface, consequently the vulcanized plate will not fit up to the palatine surface.

Dr. Land: When I make a set of teeth I invariably cover the cast with tin-foil and make it perfectly smooth just like a mirror. Then I pack the rubber not in excess on the alveolar ridge, as it is important you should know when there is enough rubber there. Then I lay on the rubber a piece of the starched cloth that comes on the sheets of rubber so as to be able to separate the flask for examination. In place of bringing the flask down with the ordinary force, where there is not enough rubber put into the flask to fill it, I put in a slight excess and close the fllask in boiling water allowing the excess to escape to the center of the mold

Separate the flask and you will find that in place of forcing the rubber to the outside you have closed the outside and have used the center for the escape and you will find that it it is filled with no excessive pressure in the center. No denture is injured unless you make excessive pressure on the center. If you do make pressure on the outside it does no harm unless it should shrink a little.

A flat mouth is not nearly so easily injured as a high arch, because if you fill the center with rubber you are going to expand it both ways in the center, there is absolutely no guess work about it at all. I always know what I am getting for when the denture is vulcanized it comes out as bright as a mirror.

Dr. Prothero: Dr. Land's method is a valuable one, but I can get just as good results by having the case carved if necessary, not putting in any excess at all before the flask is closed. I can get just as good results as he does by his forcing the vulcanite to the center, as he does. It is a question of care.. You soften plaster by enclosing it in boiling water, but you put a dry plaster cast in water and let it lay there 30 seconds and you can cut it very easily where you could not cut it before.

A visitor—I would like to say that during the last few weeks I have seen a full upper and lower made on the lines that Dr. Prothero has given us this evening, and the results were most satisfactory. I saw the plate the next morning after it had been placed and it was simply impossible to remove it unless you used pressure. The model was straight all around on the periphery and the plaster was poured as he has suggested.

I agree with Dr. Prothero that the dry heat gives the better system. Sometimes I use the moist heat, but ofttimes with the most serious results, I might say three times out of five. One thing I cannot quite understand is the way in which Dr. Hoff has suggested the removal of the extra rubber without any vents. If he has all the surplus coming to the outside of the flask I don't see how he gets rid of it. Of late years I have not used vents, except on the inside but I have had them separated so that there was as little pressure as possible and in that way obviated the running of the rubber into the embrasures. I think if all of us should pay more attention to the rubber dentures we would elevate rubber work and our patients would be better served and our conscience might possibly be a little easier. I have been using for years, almost exclusively, the metal dentures. Of late years I have been using gold nearly altogether. Formerly I used aluminum and cast dentures on the lower, but I like gold better in all cases where I can get the patient to allow me to use it.

I might say in regard to the Association, if it is not too late, I think you cannot do too much along those lines. For the last eight years, nearly, I have been in practice in South Australia, where there were about 50 dentists, but no organization. There was no organization throughout the state at all and no professional feeling. There was great jealousy. You can hardly get together half a dozen dentists there and have them friendly. I went there from California and Honolulu, where I have had the benefits off Association.

I have been a member of the California State Dental Association ever since '84 and going abroad I missed those associations more than anything else. When I first went to this place I started in with a few and tried to get them interested but I found within a year that I had made a mistake. I was continually running up against snags. I could get no one to corroborate with me and there was continual backbiting, so I kept to myself. After the experience I have had there, I feel sure that you cannot make any mistake in pushing this to the utmost.

Dr. Hoff: To prevent the red rubber running through the pink after investing the plate in the wax form in the plaster, make your investment only to the border of the plate and then when you pack your pink rubber pack it where you want it and cover it over with red rubber. and the pink rubber will not run out.

DR. HINES: In investing your model set it high in the first half of the flask, that will bring it so that the ring of the flask in the second half will come well over and keep the plaster from fracture. If the plaster surrounding the model does not fracture in some way it will not let the pink rubber be forced out. This can be obviated by investing the model high in the first half of the flask, and when you come to reverse the flask the part that you pack in will be well surrounded.

